# Scale down and optimized automated production of [68Ga]68Ga-DOTA-ECL1i PET tracer targeting CCR2 expression

**DOI:** 10.1186/s41181-023-00188-1

**Published:** 2023-02-02

**Authors:** Silvia Migliari, Maura Scarlattei, Giorgio Baldari, Livia Ruffini

**Affiliations:** Nuclear Medicine Division, Azienda Ospedaliero-Universitaria of Parma, Via Gramsci 14, 43126 Parma, Italy

**Keywords:** [68Ga]68Ga-radiopharmaceuticals, [68Ga]68Ga-DOTA-ECL1i, CC chemokine receptor type 2 (CCR2), PET imaging

## Abstract

**Background:**

Recently it has been identified a short peptide that showed allosteric antagonism against C–C motif chemokine receptor 2 (CCR2) expressed on inflammatory monocyte and macrophages. A 7-d-amino acid peptidic CCR2 inhibitor called extracellular loop 1 inverso (ECL1i), d(LGTFLKC) has been identified and labeled to obtain a new probe for positron emission tomography in pulmonary fibrosis, heart injury, abdominal aortic aneurysm inflammation, atherosclerosis, head and neck cancer. Our goal was to develop, optimize and validate an automated synthesis method for [68Ga]68Ga-DOTA-ECL1i to make it available for a broader community. The synthesis of [68Ga]68Ga-DOTA-ECL1i was done using the Scintomics GRP^®^ module with the already estabilished synthesis template for [68Ga]68Ga-DOTATOC/[68Ga]68Ga-PSMA. The radiopharmaceutical production was optimized scaling down the amount of DOTA-ECL1i (from 50 to 10 μg), evaluating synthesis efficiency and relevant quality control parameters in accordance with the European Pharmacopeia.

**Results:**

Best results were yielded with 20 μg DOTA-ECL1i and then the process validation was carried out by producing three different batches on three different days obtaining an optimal radiochemical yield (66.69%) as well as radiochemical purity (100%) and molar activity (45.41 GBq/µmol).

**Conclusions:**

[68Ga]68Ga-DOTA-ECL1i was successfully synthesized and it is, thus, available for multi-dose application in clinical settings.

**Supplementary Information:**

The online version contains supplementary material available at 10.1186/s41181-023-00188-1.

## Background

Primary focus of imaging in the era of precision medicine has moved from detection and diagnosis to characterization, prognosis and prediction of treatment. Recent years have witnessed the rapid development of novel radiopharmaceuticals, as high-resolution imaging tools, able to target specific disease hallmarks for patient stratification and treatment selection in non communicable diseases (NCDs), including inflammatory diseases.

In the western world, inflammatory diseases represent the third most common category of pathologies, after cancer and cardiovascular diseases, and they are caused by a self-harm process carried out by the immune system and involve monocytes and their lineage-descendant macrophages.

Very often the reason for this self-injury is not known, but the regulation of the products, the recruitment, the activation, or the differentiation of the monocyte pool is a viable therapeutic strategy to fight chronic or acute inflammatory disease (Geissmann et al. 2010; Gordon and Taylor 2005). Recruitment of inflammatory monocytes is mainly driven by CC chemokine receptor type 2 (CCR2) and one of its ligands, CC chemokine ligand type 2 (CCL2) (Charo et al. 1994). Accordingly, the critical role of CCR2/CCL2 in various inflammatory pathogeneses, including rheumatoid arthritis (Bayley et al. 2003; Quinones et al. 2004), asthma (Blease et al. 2000; Rose et al. 2003), multiple sclerosis (Izikson et al. 2000; Fife et al. 2000; Huang et al. 2001), neuropathic pain (Abbadie et al. 2003), or atherosclerosis (Boring et al. 1998), has been largely documented and demonstrated in mice that are deficient for CCL2 or CCR2. These studies provide strong evidence that CCR2 is a promising clinical drug target in the treatment of inflammatory diseases but the developed CCR2 antagonists have failed in a lot of phase II clinical trials, due to the lack of efficacy, showing undesired adverse effects (e.g. cardiovascular liabilities and citotoxity) (Bachelerie et al. 2014; Auvynet et al. 2016). Most of these molecules New approaches going beyond these obstacles have exploited a different binding pocket, called “allosteric site”, and using peptidic non-competitive antagonists (Bachelerie et al. 2014).

These new molecules produce a non-competitive effect, being directed towards the G-protein-coupled receptors’ (GPCRs) extracellular loops, which have a critical role in the process of receptors conformational modification. Particularly for CCR2, many studies refer to a small allosteric peptidic modulator. An original 7-d-amino acid peptidic CCR2 inhibitor called extracellular loop 1 inverso (ECL1i), d(LGTFLKC) that targets the juxtamembranous extracellular loop 1, called Extracellular Loop 1 Inverso (ECL1i), reducing the interaction with orthosteric agonist and also avoiding the various undesired effects (Auvynet et al. 2016).

The identification of this new target has been useful in the formulation of a novel tracer for specific non-invasive molecular-targeted imaging with positron emission tomography (PET) (Auvynet et al. 2016; Heo et al. 2019).

ECL1i has been functionalized with DOTA chelator to obtain radiolabeled probes for PET imaging in humans with pulmonary fibrosis (Brody et al. 2021), cardiovascular diseases [NCT05107596, NCT04592991, NCT04537403], head and neck cancer [NCT04217057] and for predicting response to CCR2-Targeted therapy in pancreatic cancer [NCT03851237].

The DOTA-ECL1i moiety has also been used to quantify CCR2-expressing cells in mouse models and human tissues of lung inflammation (Liu et al. 2017), cardiac injury (Heo et al. 2021; English et al. 2020), cardiac and tissue remodeling (Cifarelli et al. 2022; Gallerand et al. 2021).

Radionuclides used for the radiolabeling are gallium-68 and cupper-64, and 64Cu-DOTA-ECL1i is reported in the NCI Drug Dictionary as a PET tool for quantification of CCR2-expressing cells (https://www.cancer.gov/publications/dictionaries/cancer-drug/def/copper-cu-64-dota-ecl1i).

However, gallium-68 is more suitable for routine procedures because of the on-site production from generators, the high price and insufficient commercial availability of Cu-64 (Fani et al. 2008; do Carmo et al. 2020) and the efforts spent by our group in developing and validating [68Ga]68Ga-based targeted tracers gallium-68 for PET imaging in the clinical and research context (Migliari et al. 2022a, 2022b, 2021, 2017; Sammartano et al. 2022).

The aim of this work was to develop a synthesis automated method to label extracellular loop 1 inverso (DOTA-ECL1i) with gallium-68 (Additional file 1: Fig. S1) and a quality control (QC) system to determine its radiochemical purity.

## Results

### Labelling and quality controls results of different DOTA-ECLI1 loads and of the validated synthesis

The fully automated production of [68Ga]68Ga-DOTA-ECL1 was conducted investigating five different precursor amounts of DOTA-ECL1i (10–20–30–40–50 μg) for radiolabelling with gallium-68. Immediately after each synthesis the overall QCs of the final product (Table 1) were performed in order to determine the best precursor amount from which to start to obtain [68Ga]68Ga-DOTA-ECL1 and to optimize the entire production process.

**Table 1:** Summary data of [68Ga]68Ga-DOTA-ECL1 QCs (10–50 μg, n = 3)

Peptide (DOTA-ECL1i, ug)	50 (250 μL, 0.038 μmol)	40 (200 μL, 0.031 μmol)	30 (150 μL, 0.023 μmol)	20 (100 μL, 0.015 μmol)	10 (50 μL, 0.0076 μmol)
Radiochemical purity (Radio-UV-HPLC)	100%	100%	100%	100%	100%
Radiochemical purity (Radio-TLC)	100%	100%	100%	100%	100%
pH	7	7	7	7	7
Radiochemical yield (n.d.c.)	55.90%	54.03%	62.45%	66.69%	53.21%
Volume	10	10	10	10	2–10 mL
Color	Colorless	Colorless	Colorless	Colorless	Colorless
Molar activity	15.026 GBq/µmol	16.51 GBq/µmol	30.34 GBq/µmol	45.41 GBq/µmol	71.51 GBq/µmol

The best results were obtained for 20 μg of precursor, therefore once the automated synthesis has been optimized, the production processes have been validated with this amount of peptide precursor, according to regulatory requirements to warrant the robustness of the gallium-68 labelling methods of DOTA-ECL1i. Some tested QC parameters were based on the European Pharmacopoeia (11.0/0125) (Table 2).

**Table 2:** Summary data of three consecutive validation batches of [68Ga]68Ga-DOTA-ECL1 (20 μg)

Test	Batch 1	Batch 2	Batch 3	Acceptance criteria
Radiochemical purity (Radio-UV-HPLC)	100%	100%	100%	> 95%
Radiochemical purity (Radio-TLC)	100%	100%	100%	> 95%
pH	4–8.5	7	7	4–8.5
Radiochemical yield (n.d.c.)	63.00%	63.00%	66.00%	> 40%
Radioactivity concentration	75.6–52.48	75.6–52.48	79.2–54.98	> 50 MBq/mL
Radioactivity	756–524.79	756–524.79	792–549.78	> 150 MBq
Volume	10	10	10	2–10 mL
Color	Colorless	Colorless	Colorless	Colorless
Molar activity	42.89 GBq/µmol	42.89 GBq/µmol	44.94 GBq/µmol	1–60 GBq/µmol
Radionuclidic purity	100%	100%	100%	> 99.9%
Ge-68 breakthrough	0.00000035%	0.00000033%	0.00000035%	< 0.001%
EtOH amount	3.73%	3.69%	3.43%	< 10% (V/V) (< 2.5 g)
HEPES content	9.45 μg/mL	9.45 μg/mL	9.45 μg/mL	Less than 200 µg/V of HEPES in test solution
Endotoxins	< 17.5 IU/mL	< 17.5 IU/mL	< 17.5 IU/mL	< 17.5 IU/mL
Sterility test	Sterile	Sterile	Sterile	Sterile
Stability over 3 h (RCP%)	100%	100%	100%	> 95%

The radiochemical purity (RCP% = 100%-colloids-ions) was evaluated verifying the prence of free gallium (based on Radio-UV-HPLC) and gallium colloids (based on Radio-TLC).

**Fig. 1:**
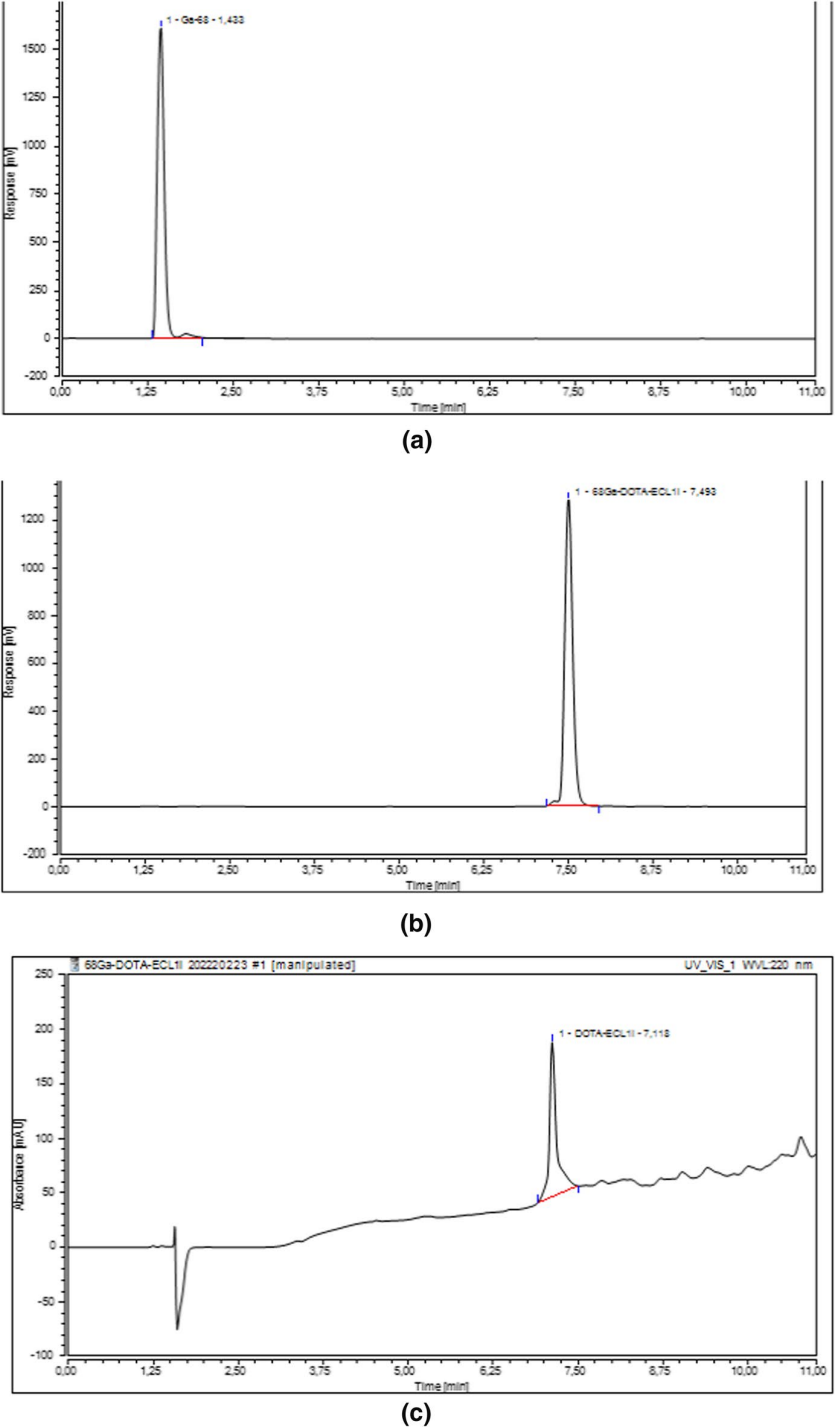
**a** HPLC chromatogram of the radiodetector showing the eluate [68Ga]68GaCl_3_; **b** HPLC chromatogram of the radiodetector showing [68Ga]68Ga-DOTA-ECL1. The small minor peak just before [68Ga]68Ga-DOTA-ECL1 belongs to [68Ga]68Ga-DOTA-ECL1peak, infact in UV chromatogram there is only the peak of DOTA-ECL1i (Fig. 1c); **c** Radio-UV-HPLC chromatogram of DOTA-ECL1i

With Radio-UV-HPLC, free gallium-68 was detected at Rt = 1.433 min (Fig. 1a), whereas gallium-68 bound to DOTA-ECL1i was detected at Rt = 7.493 min (Fig. 1b) with a purity of 100%.

The reference solution of DOTA-ECL1i showed a little bit different retention time (Rt = 7.118 min), as seen in Fig. 1c, respect to [68Ga]68Ga-DOTA-ECL1 Rt = 7.493 min. The difference of the two retention times is due to the use of different detectors (Radio and UV–VIS), but also due to a different charge of the DOTA chelator after incorporation of gallium-68. The different charge results in changed interaction with the column due to a slight change of hydrophobicity of the complete molecule. The absence of 68Ga-chelate in the standard solution, maintains 3 of free carboxylic acid causing the increased hydrophilicity of the standards and then modifying its Rt.

**Fig. 2:**
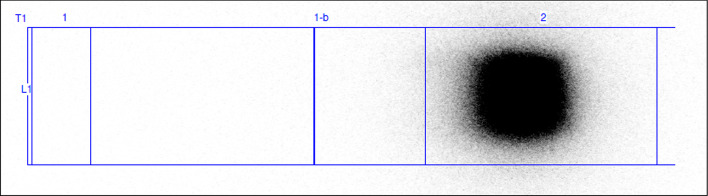
Radio-TLC chromatogram of [68Ga]68Ga-DOTA-ECL1

With Radio-TLC no [68Ga]68Ga-colloids could be detected at R_f_ = 0.2 and the radiopharmaceutical product was detected at Rf = 0.8 (Fig. 2).

In addition the product was tested for endotoxins and their concentration resulted below 17.5 EU/mL for all samples. Sterility was assessed in all samples that resulted sterile.

**Fig. 3:**
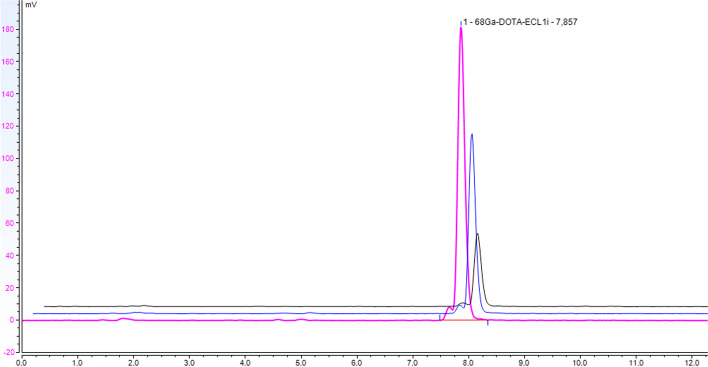
Stability of [68Ga]68Ga-DOTA-ECL1 (pink dash: 1 h, blue dash: 2 h, black dash: 3 h)

The stability of [68Ga]68Ga-DOTA-ECL1 in buffer solution at room temperature was tested up to 3 h by Radio-UV-HPLC, RadioTLC and pH. As seen in Fig. 3 [68Ga]Ga-DOTA-ECL1i is stable under the setup conditions. No additional radioactive by products or free gallium-68 could be detected in this time period and the RCP% stays 100% over time.

**Fig. 4:**
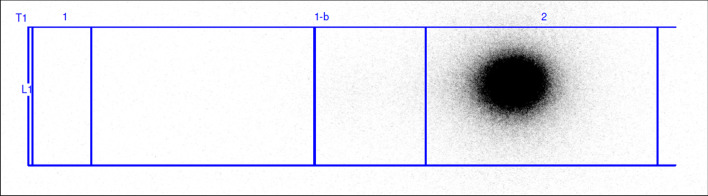
Radio-TLC chromatogram of [68Ga]68Ga-DOTA-ECL1 over 3 h

RCP% was assessed and confirmed also with radio-TLC (Fig. 4), meanwhile pH value stays 7 over 3 h.

## Discussion

The key functions of CCR2 in inflammatory monocyte recruitment, and, thus, in such inflammatory diseases, make this receptor an active target for drug discovery and development. We hereby describe the development and validation of an automated synthesis method and QC system to label with gallium-68 the first potent allosteric, noncompetitive, peptidic inhibitor of CCR2, ECL1i functionalized with DOTA chelator.

The development-design process of a new radiopharmaceutical ordinarily involves the building and the setup of the radiosynthesis as well as the quality assessment methods of the final product through the evaluation of release specifications as RCP%, A_s_ or A_m_, radionuclidic purity, chemical purity, radiochemical yield (RCY%), pH, sterility and stability.

One of the parameters of paramount importance is the specific activity or molar activity in the final product, i.e. the ratio of the labeling isotope (in Bq) divided by the amount of the peptide (g) or the unit mole of the compound (mol).

For the synthesis setup optimization, molar activity (A_m_) is required to be high due to several biological factors, such as the affinity of the peptide for its receptor and the number of available receptors, limiting the mass dose of peptide that can be administered. Indeed, a suboptimal A_m_ could result in excessive amount of peptide leading to the saturation of the receptor or eventually to the induction of pharmacological side effects.

On the other hand, A_m_ should not be too high because the amount of the peptide should be sufficiently high to allow reasonable radiochemical yield and to guarantee a reliable biodistribution for image quantification.

For these reasons, a scale down of five different amounts of DOTA-ECL1i (50–40–30–20–10 μg) was evaluated for the production of [68Ga]68Ga-DOTA-ECL1. The results summarized in Table 1 demonstrate that the precursor amount of 20 μg allows the best radiochemical yield (66.69%) as well as high radiochemical purity (100%) and good molar activity (45.41 GBq/µmol).

We noticed a decreasing radiochemical yield corresponding to increasing ligand amount (higher RCY% for 20 and 30 µg, and lower for 40 and 50 µg), (Table 1).

The phenomenon could be caused by either the possible presence of impurities in the precursor peptide or by the steric hindrance of the molecule.

However, the chromatogram of the GMP-grade precursor DOTA-ECL1i doesn’t show any impurity (Fig. 1c, page 8) and the solutions of precursor used for labeling belong to the same mother solution obtained dissolving the peptide with metal free water without further dilutions. In addition, the purification of eluate on PS-H + allow to avoid the possible ion interference during labelling reaction. Therefore, the decreased RCY% could be caused by the higher steric hindrance of a higher amount of peptide.

A turning point is the selection of the ideal lowest precursor amount guaranteeing high RCP%, RCY% and A_s_ as well as avoiding adverse effects.

The use of the same small volume of gallium-68 eluate with higher concentration allows, for a reduced amount of ligand, a faster radiolabeling reaction with quantitative incorporation and high tracer specifc activity (Nelson et al. 2022; Velikyan 2015).

Therefore, the optimization of the radiopharmaceutical production led us to compare results from 10 and 20 μg of DOTA-ECL1 for radiolabelling.

The obtained lower radiochemical yield for 10 μg with elevated RCP% value and higher molar activity suggest that this amount is probably too much low for optimal complexation with gallium-68, leading us to validate the radiosynthesis of [68Ga]68Ga-DOTA-ECL1 with 20 μg of DOTA-ECL1i.

Harmonisation and standardisation of radiopharmaceutical production is necessary to allow radiopharmaceutical research to be unambiguously and methodologies tested and transferred between laboratories. Once the method has been adequately optimized, the process and the final product was validated (Table 2), a regulatory requirement, to warrant the robustness of the gallium-68 labelling method of DOTA-ECL1i, ensuring clinical suitability and adherence to well-defined standard operation procedures.

The results of all tested quality control parameters demonstrate and confirm the high reproducibility of the [68Ga]68Ga-DOTA-ECL1 production method resulting safe and readily available, enabling the daily production and transferability of it in other radiopharmaceutical laboratories.

Next step will be the preclinical assessment of [68Ga]68Ga-DOTA-ECL1 to detect a distorted expression of CCR2 identifying the consequential pathologies for a future implementation in clinical practice.

## Conclusions

[68Ga]68Ga-DOTA-ECL1 was successfully synthesized in a fully-automated way on GRP Scintomics module. All the tested quality parameters for radiochemical purity, pH, endotoxins and sterility were in accordance with European Pharmacopoeia. In addition the product solution was stable for at least 3 h after production, as shown by Radio-UV-HPLC. Thus, [68Ga]68Ga-DOTA-ECL1 can be easily and reliably produced for routine clinical application.

## Material and methods

### Reagents and solvents

All chemicals used for the radiolabelling reaction (saline, ethanol, HEPES buffer solution and water) were of the highest available purity grade and commercially obtained as a single disposable kit (reagents and cassettes for synthesis of [68Ga]68Ga-peptides using cationic purification ABX, Advanced Biochemical Compounds, Radeberg, Germany).

The GMP peptides DOTA-ECL1i was purchased as lyophilized powder from piCHEM (Forschungs- und Entwicklungs Grambach, Austria).

An aqueous mother solutions of DOTA-ECL1i (200 μg/mL) was prepared in ultrapure water (Sigma Aldrich), kept at − 20 °C and the different solutions of precursor used for labeling belong to the same mother solution.

Gallium-68 (t1/2 = 68 min, β +  = 89%, and EC = 11%) was obtained from a pharmaceutical grade 68Ge/68Ga generator (1850 MBq, GalliaPharm^®^ Eckert & Ziegler, Berlin, Germany) by elution with 0.1 M HCl (Rotem GmbH, Germany). The amount of detected metal impurities as provided by the manufacturer was less than the defined limit in the European Pharmacopeia monograph (Chloride and (68Ga) solution for labeling (Monograph 2464) 2464).

The reagents trifluoroacetic acid (TFA), water and acetonitrile used for Radio-UV-HPLC, as well as ammonium acetate and methanol were metal free and purchased from Sigma Aldrich (Saint Louis, Missouri, USA). The aseptic production was conducted in a GMP grade A hot cell (NMC Ga-68, Tema Sinergie). Both ^68^Ge/^68^Ga generator and automated synthesis module (Scinotmics GRP^®^ module, Germany) were placed in the hot cell.

### Preparation for labelling of DOTA-ECL1i with gallium-68

First the commercially available fully automated synthesis platform Scinotmics GRP^®^ module was equipped with a disposable single-use cassette (SC-01, ABX).

DOTA-ECL1i was prepared in 10–20–30–40–50 μg aliquots from the stock solution (200 μg/mL). All synthesis reagents were contained in the reagent set. From the reagent set, a syringe containing 1.5 mL of 5 M NaCl was attached into the cassette as well as phosphate buffer saline (PBS), ethanol and ethanol/water 1/1 vials in each designated valve.

Cation exchange (SCX) cartridge and C18 column (Sep Pak C18 RP) were inserted into appropriate holders into the cassette and module.

The reaction mixture containing 3.2 mL 1.5 M 4-(2-hydroxyethyl)-1-piperazineethanesulfonic acid (HEPES) buffer solution and an aliquot of DOTA-ECL1i was loaded into the reaction vial.

### Labelling of DOTA-ECL1i with [68Ga]68GaCl_3_

The synthesis was performed fully-automated and each reaction parameter as reaction time, temperature and radioactivity was monitored in real time. Gallium-68 was obtained eluting a GMP certified 68Ge/68Ga generator (GalliaPharma^®^, Eckert and Ziegler) with 0.1 M HCl, collected and pre-purified on a strong cation exchange (SCX) cartridge which is then eluted with 5 M NaCl.

The eluate is added into the reaction vial, previously loaded with DOTA-ECL1i (10–20-30–40–50 μg in 1.5 M 2-[4-(2-hydroxyethyl)-1-piperazinyl]-ethanesulfonic acid (HEPES buffer solution) at pH = 4–4.5. The mixture was incubated at 95 °C for 10 min.

After the completion of labelling reaction the crude product was cooled down and trapped onto Sep Pak C18 RP cartridge, washed with water for injection Ph. Eur., and eluted with 2 mL of Ethanol/Water 1/1.

**Fig. 5:**
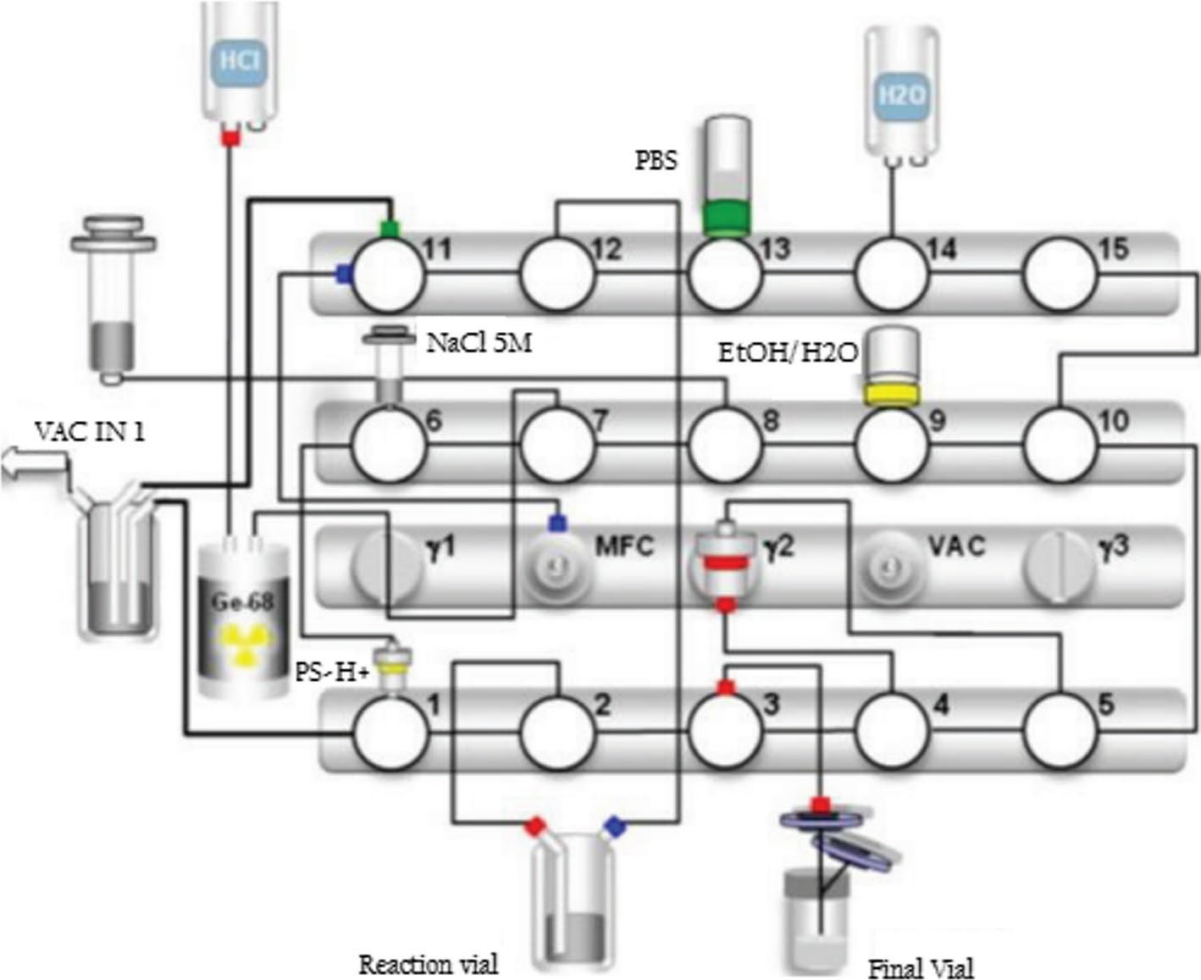
Synthesis scheme of automated Scintomics module GRP^®^ to produce [68Ga]68Ga-DOTA-ECL1

The final product was diluted with phosphate buffered saline (PBS) and sterilized through a 0.2 μm filter (millex GV) into a sterile 25 mL capped glass vial and diluted with PBS for the final formulation (Fig. 5). The entire radiopharmaceutical production takes 35 min.

### Quality control and process validation

After synthesis, the radiopharmaceutical product was evaluated for quality control determining the following parameters: total product activity, gallium-68 ion identity via half-life time and gamma spectroscopy, chemical and radiochemical purity by Radio-UV-HPLC and Radio-TLC, pH, radionuclide purity for 68Ge-breakthrough and sterility/endotoxin assay (sterility test and LAL test).

Radiochemical purity (RCP) is one of the most important quality criteria to release the final product for the clinical use, as described in the European Pharmacopeia (Decristoforo et al. 2017; Gillings et al. 2021; Todde et al. 2017; Blois et al. 2019). To this aim, a fully validated separation method has to be available according to ICH guidlines (ICH 2017), enabling optimal separation between different (radio) chemical forms (radioactive impurities) other than the original intact radiopharmaceutical (ICH guideline Q2(R1) 2005; Levin 2010; Zarghi et al. 2011).

Moreover, the stability of [68Ga]68Ga-DOTA-ECL1 at room temperature was monitored by Radio-UV-HPLC for 3 h. To validate the entire process of radiopharmaceutical production and quality control, three batches of [68Ga]68Ga-DOTA-ECL1 were produced in three different days under the same conditions set for typical routine preparations. Every batch was fully characterized from the analytical point of view, with the aim to verify that the product meet the acceptance criteria for all the established quality parameters.

#### Appearance

The visual inspection of in-house prepared radiopharmaceuticals is necessary before injection into the patient, as a measure of process performance and validation. Presence of particulate in the sample suggests possible failure during radiopharmaceutical synthesis, including purification, sterilizing filtration, and failed environmental control during the setting-up of reagents (ICH 2017).

#### Instant thin layer chromatography

Radio-TLC test was used to determine the percentage of [68Ga]68Ga-DOTA-ECL1 and gallium-68 colloids in the final product.

For the determination of gallium-68 colloids amount, 1 M ammonium acetate/methanol (1:1 vol/vol) was used as mobile phase and ITLC-SG paper strips (Varian ITLC-SG plates) as stationary phase; gallium-68 colloid (Rf = 0.0–0.2), [68Ga]68Ga-DOTA-ECL1 (Rf = 0.8–1).

TLC-SG paper strips, used a stationary phase, were counted with a scanner (Cyclone^®^ Plus Storage Phosphor system, Perkin Elmer) and the chromatograms were analysed with OptiQuantTM software.

#### High pressure liquid chromatography

The Radio-UV-HPLC was additionally used to determine the chemical and radiochemical purity of [68Ga]68Ga-DOTA-ECL1 in the final product after the purification with C18 cartridge, at the end of the synthesis.

Radio-UV-HPLC was performed on a Dionex Ultimate 3000 HPLC system (Thermo Fisher Scientific) equipped with a AcclaimTM 120 C18 column 3 µm 120 Å (3.0 mm × 150 mm) and a UV and a γ-detector (Berthold Technologies, Milan, Italy). The used solvents were A) 0.1% TFA in water and B) 0.1% TFA in acetonitrile.

The flow rate of the mobile phase was set at 0.6 mL/min, with a total run of 20 min.

The following phase gradient was used in the Radio-UV-HPLC analysis: 0–16 min from 0% B to 90% and 16–20 min from 90 to 0% B.

The column temperature was kept at 30 °C. The samples were also monitored with UV detector at 220 nm in order to detect chemical impurities in the final product. Activity corresponding to gallium-68 ion and [68Ga]68Ga-DOTA-ECL1 was measured by Radio-UV-HPLC γ-detector.

The software system Chromeleon 7 was used to assemble the information.

#### Ge-68 breakthrough

The Ge-68 breakthrough was measured by gamma spectroscopy of the final product, using a gamma spectrometer equipped with a high-purity germanium (HPGe detector ORTEC GEM 30P4-76). The *γ*-ray spectrometry tests included the identification of principal *γ*-photon (499–521 keV peak) and Ge-68 content (decay of 499–521 keV peak ≥ 48 h) using a large volume counter linked to a multichannel analyser system (HPGe detector ORTEC GEM 30P4-76).

The spectra acquisition of Ge-68 was performed at least 48 h after the completely decay of Ga-68 produced by the generator.

Duration of the acquisition was 180 min to obtain a high signal-to-noise ratio. The sample volume was at least 1 mL. Spectrum was analyzed using Genie 2000 software.

#### Radionuclide identification and activity measurements

Radionuclide purity was determined based on the half-lives, type and energy of the emitted radiations. Half-life was measured with a dose calibrator (Capintec 25-R) at four consecutive intervals (5, 10, 15 and 20 min). The expected half-life of gallium-68 is 67.6 min and is calculated using the following equation:

$$T1/2 = - ln2\left( {dt,ln\left( {A1/A0} \right)} \right)$$

where: dt-time difference, A1-ending activity, A0-starting activity.

#### pH evaluation

The pH value of [68Ga]68Ga-DOTA-ECL1 was measured using colorimetric pH strips (0–14).

#### Endotoxin and sterility

Quantitative determination of bacterial endotoxins was performed by the chromogenic method, using Endosafe^®^ nexgenPTS™ (Charles River, Ireland) apparatus. [68Ga]68Ga-DOTA-ECL1 samples were previously diluted and then applied in duplicate inside cartridges in parallel with positive control testing. The radiopharmaceutical can be released when the level of endotoxins was less 17.5 IU/mL in accordance with *Ph. Eur.* (9.0/0125).

The sterility of the [68Ga]68Ga-DOTA-ECL1 solution was assessed by direct inoculation in a growth broth (Triptic Soy Broth, TSB) which was incubated at 20–25 °C, and verified daily over fourteen days (Guidelines 2018). The sample was considered sterile when no microbiological growth was detected.

#### Residual solvents and HEPES

Potentially present radiolysis products, such as ions and excited molecules could cause undesired and serious side effects (Gillings et al. 2020; International Atomic Energy Agency (IAEA)/World Health Organization (WHO) guideline on good manufacturing practices for investigational radiopharmaceutical products, Draft working document 2021,2021; ; ; ; ). Radiolysis may be reduced by utilizing compounds insensitive to radiation or extenuating the process with additives (e.g. radical scavengers). In the clinical context, these radical scavengers should be suitable for human use, such as ascorbic acid or HEPES in the reaction mixture and ethanol in the pre- and post-processing steps. The positive influence of ethanol on radiolabeling yield and radiolysis restrain (Annex 2 International Atomic Energy Agency and World Health Organization (WHO) guideline on good manufacturing practices for radiopharmaceutical products 2020,2020; International Atomic Energy Agency (IAEA)/World Health Organization (WHO) guideline on good manufacturing practices for investigational radiopharmaceutical products, Draft working document 2021,2021) prompted us to use it during the synthesis process in order to obtain a more reliable and repeatable automated method.

Ethanol is a class 3 solvent which may remain indeterminate and unmentioned up to 0.5%, but must be declared quantitatively for higher amounts in a pharmaceutical (Ph. Eur. 2019, 9.6. 9.7–9.8).

In this study we determined the residual ethanol using gas chromatography (GC) and HEPES content by TLC-SG, both according to *Ph. Eur.* Monograph (Chloride and (68Ga) solution for labeling (Monograph 2464) 2464). The reference solution of HEPES was prepared at a concentration of 200 µg/mL. Two separate spots of reference solution (5 µL) and test solution (a sample of the final product [68Ga]68Ga-DOTA-ECL1 were applied on TLC silica gel F_254_ plate and developed on a path over 2/3 of the plate with use of water: acetonitrile (25:75 V/V) solution as a mobile phase. The plate was then exposed to iodine vapor. The spot corresponding to the test solution should not be more intense than the reference solution spot (less than 200 µg/V of HEPES in test solution). HEPES content has been assessed also using the validated HPLC method (Migliari et al. 2022a) based on the use of a Waters Xbridge^®^ column C18 (150 mm × 4.6 mm, 3.5 μm), as stationary phase, connected to an UV detector set to a wavelength of 195 nm and a γ-detector (Berthold Technologies, Milan, Italy) and ammonium formate 20 mM pH 9.5, as mobile phase, at an isocratic flow of 0.7 mL/min.

## Supplementary Information

**Additional file 1: Fig. S1.** [68Ga]68Ga-DOTA-ECL1i radiopharmaceutical. Chemical structure of [68Ga]68Ga-DOTA-ECL1i

## Data Availability

All data generated and analysed during this study are included in this published article.

## Abbreviations

GMP: Good manufacturing practice
GRP: Good radiopharmaceutical practices
GC: Gas chromatography
HPLC: High pressure liquid chromatography
ICH: International Conference of Harmonisation
LAL test: Limulus amebocyte lysate test
QC: Quality control
PET: Positron emission tomography
TLC: Thin layer chromatography
TFA: Trifluoroacetic acid
