# Supplementary figures and images for: Scale down and optimized automated production of [68Ga]68Ga-DOTA-ECL1i PET tracer targeting CCR2 expression
Source: EJNMMI Radiopharm Chem. 2023 Feb 2;8:3. doi: 10.1186/s41181-023-00188-1 (PMC9895323; doi:10.1186/s41181-023-00188-1)

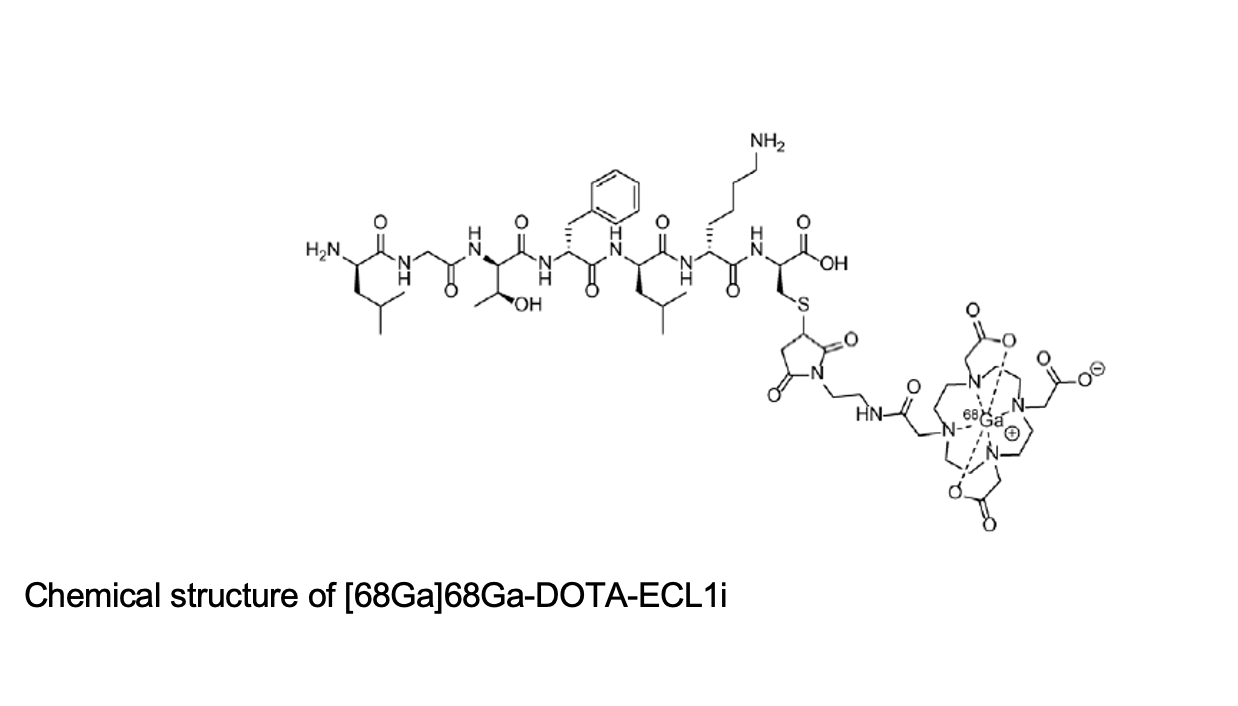

Supplement: Supplementary file 1 — Additional file 1: Fig. S1. [68Ga]68Ga-DOTA-ECL1i radiopharmaceutical. Chemical structure of [68Ga]68Ga-DOTA-ECL1i [file 41181_2023_188_MOESM1_ESM.png]
